# Combination of *Cyclamen persicum* Mill. floral gene promoters and chimeric repressors for the modification of ornamental traits in *Torenia fournieri* Lind.

**DOI:** 10.1038/hortres.2017.8

**Published:** 2017-03-22

**Authors:** Ichiro Kasajima, Norihiro Ohtsubo, Katsutomo Sasaki

**Affiliations:** 1 Institute of Vegetable and Floriculture Science, National Agriculture and Food Research Organization (NARO), Ibaraki 305-8519, Japan

## Abstract

Although chimeric repressors such as the Arabidopsis TCP3 repressor are known to have significant effects on flower morphology and color, their cellular-level effects on flower petals are not understood. The promoter sequences of the genes expressed in the flowers of cyclamen, a representative potted flower grown during the winter season, are also unknown. Here, we isolated eight promoters from cyclamen genes that are reportedly expressed in the petals. These promoters were then fused to four chimeric repressors and introduced into the model flower torenia to screen for effective combinations of promoters and repressors for flower breeding. As expected, some of the constructs altered flower phenotypes upon transformation. We further analyzed the effects of chimeric repressors at the cellular level. We observed that complicated petal and leaf serrations were accompanied by excessive vascular branching. Dichromatism in purple anthocyanin was inferred to result in bluish flowers, and imbalanced cell proliferation appeared to result in epinastic flowers. Thus, the genetic constructs and phenotypic changes described in this report will benefit the future breeding and characterization of ornamental flowers.

## Introduction

The breeding of new flower cultivars with various phenotypes, such as different petal colors and shapes, is key to promoting flower use. Straightforward destruction of endogenous genes, introduction of new metabolic pathways from different species through transformation, and random mutagenesis using ion beam irradiation have been effective in generating double flowers as well as altering flower color, pigmentation patterns, and shapes (for example, refs 1–5). In addition to these approaches, the use of chimeric repressors to confer different characteristics upon flowers in a dominant genetic manner has also been tested.

Chimeric repressors are artificially generated by combining transcription activators with peptides known as repression domains. In plants, class II ethylene-responsive element-binding factor and TFIIIA-type zinc finger repressors, including SUPERMAN, share the ‘EAR motif’.^6^ Short peptides that include this motif function as repression domains, changing transcription activators into transcription suppressors. The most frequently used and strongest repression domain is ‘SRDX,’ which is a leucine-rich peptide consisting of 12 amino acid residues (LDLDLELRLGFA). This peptide was obtained by altering the native sequence of the repression domain of SUPERMAN.^7,8^ The wide range of flower phenotypes generated to date using chimeric repressors is listed in the FioreDB database (http://www.cres-t.org/fiore/public_db/index.shtml; refs 9,10).

Due to the ease of transformation compared with other ornamental crops, torenia (*Torenia fournieri* Lind.) is used as a model system for transgenic flower breeding. Various chimeric repressors of Arabidopsis (*Arabidopsis thaliana* (L.) Heynh.) transcription factors have been surveyed in torenia.^11^ According to the comparison of all such chimeric repressors examined in torenia thus far, repressors of *TCP* (Teosinte branched1, Cycloidea and Pcf) and *SEP3* (SEPallata3) are among those causing the clearest phenotypic changes.^12–15^ Endogenous promoters of torenia have been used to drive the Arabidopsis TCP3 repressor to change torenia flower phenotypes specifically and in various ways.^16^ Thus, variation in promoters and chimeric repressors promotes the breeding of a variety of transgenic flowers. However, despite the publication of several relevant reports, the physiological effects of chimeric repressors linked to phenotypic changes in transgenic flowers, such as petal serration, have not been examined in ornamental flowers.

In the present study, we first isolated promoters from cyclamen (*Cyclamen persicum* Mill.), which is a representative potted winter-flowering plant. We identified eight promoter sequences of genes known to be expressed in cyclamen petals. We then cloned these promoters and used them to drive four different chimeric repressors in torenia. Examination of the transgenic torenia flowers harboring these genetic constructs allowed us to characterize the effects of these cyclamen promoters and chimeric repressors. Further analyses of the transgenic lines suggested morphological or biochemical effects linked to the phenotypic changes in the flowers.

## Materials and methods

### Isolation of cyclamen promoters

The full-length cDNA sequences of *CpAP1*, *CpPI*, *CpAP3A*, *CpAP3B*, *CpSEP2*, *CpSEP3*, *CpFLC*, *CpCHS*, *CpDFR* and *CpOMT* were previously reported (Table 1). Three gene-specific primers (GSP1, GSP2 and GSP3; Supplementary Table S1) were designed in reverse orientation corresponding to the 5′-untranslated region (UTR) or the first exon of each cDNA sequence, and additional gene-specific primers were designed for some of the genes to extend the promoter sequences. Genomic DNA was extracted from cyclamen (*Cyclamen persicum* Mill. cultivar ‘Mini Loyal Purple’) leaves using ‘PVPP buffer’ containing 4 m NaCl and was purified via ultracentrifugation.^17,18^ The promoter sequences were amplified by adaptor PCR with a ‘mismatched DS4 adaptor’ (ref. 19; Supplementary Table S1), which potentially has a better capability to amplify target sequences. Briefly, genomic DNA was digested with eight different restriction enzymes (*Dra*I, *Eco*RV, *Sca*I, *Ssp*I, *Hin*dIII, *Kpn*I, *Sac*I and *Spe*I) and then ligated to adaptors and amplified by nested PCR using gene-specific primers and adaptor primers (DS4-AP1, DS4-AP2 or DS4-AP3; Supplementary Table S1). PCR was performed with KOD Plus Neo polymerase (Toyobo, Osaka, Japan) for 35 cycles, with an annealing temperature of 68 °C and an extension time of 5 min. The amplified fragments were recovered from the agarose gels, TA-cloned into plasmids, and sequenced. The promoters were then amplified from the genomic DNA using cloning primers (CP-F and CP-R; Supplementary Table S1) in which *Hin*dIII (5′- AAGCTT-3′) or *Bam*HI (5′- GGATCC-3′) sites were attached at either end for subcloning (underlined in Supplementary Table S1). Amplification was performed with KOD Plus Neo polymerase for 45 cycles, with an annealing temperature of 68 °C and an extension time of 5 min. The accession numbers and lengths of the isolated promoters are shown in Table 1.

### Subcloning

The protein-coding regions of *AtTCP3*, *CpTCP1B*, *AtSEP3*, and *CpSEP3* (International Nucleotide Sequence Database Collaboration (INSDC) accession numbers NM_104201.3, LC186052, NM_180622.2 and AB600236.1, respectively) were cloned into the *Bam*HI*–Sma*I site of the p35SSRDXG vector^20^ to produce *35S*p:*CR* vectors. Single-nucleotide (silent) mutations were introduced into the *AtSEP3* and *CpSEP3* sequences to eliminate restriction sites without changing the encoded protein sequences. Cyclamen promoter fragments were introduced into the *Hind*III-*Bam*HI sites of *35S*p:*CR* vectors or into the pG2-35S-GUS vector to produce 32 *CPG*p:*CR* vectors and four *CPG*p:*GUS* vectors, respectively. The regions corresponding to each transgene were transferred into the binary vector pBCKH (for hygromycin selection of transgenic plants) or pBCKK (for kanamycin selection of transgenic plants; ref. 20) using the Gateway system (Thermo Fisher Scientific, Waltham, MA, USA).

### Plant materials, plant growth conditions and transformation

*In vitro* cloned seedlings of torenia (*Torenia fournieri* Lind. cultivar ‘Crown Violet’ and the hybrid torenia cultivar ‘Summerwave Blue’) were maintained on sterilized media in transparent plastic boxes at 25 °C under white fluorescent light (photosynthetic photon flux density (PPFD)=150 μmol m^−2^ s^−1^) with a 16-h/8-h light/dark photoperiod. For transformation, bulk (mixture) or individual plant expression vectors carrying the appropriate constructs were introduced into *Agrobacterium tumefaciens* strain EHA105 via electroporation, and the *Agrobacterium* were subsequently infiltrated into leaf discs as previously described.^21^ Briefly, thousands of 5-mm leaf discs were prepared from *in vitro* cultures, inoculated with *Agrobacterium*, then co-cultured on media without antibiotics, and transformed calli were generated on selection media containing antibiotics. One or two transgenic lines were typically obtained from 100 leaf discs. The number of total leaf discs was ~8000. Regenerated plant cultures were maintained on media containing trehalose, and the media were renewed every 2 months.^22^ Transformants were then transferred to pots and grown in a sunlit greenhouse.

The transformants generated with bulk plasmids were examined via nested PCR to determine the inserted constructs (Supplementary Figure S4). The first round of PCR was performed with ‘Whole-F1’ and ‘Whole-R1’ primers. The first PCR product (diluted 1/1000 in solution) was then amplified using a combination of the ‘Whole-F2’ primer, individual promoter-specific primers (PSP; Supplementary Table S1), and the mixture of all repressor-specific primers (RSP; Supplementary Table S1). Both the first and second rounds of PCR were performed with KOD Plus Neo polymerase for 40 cycles, with an annealing temperature of 55 °C and an extension time of 2 min. The repressor genes (*AtTCP3-RD*, *CpTCP1B-RD*, *AtSEP3-RD* or *CpSEP3-RD*) introduced into the transformants were finally determined based on the PCR product sizes (approximately 650 bp for *CpTCP1B-RD*, 500 bp for *CpSEP3-RD*, 400 bp for *AtSEP3-RD*, and 200 bp for *AtTCP3-RD*). The whole-F2 primer generates large DNA fragments when there is no construct corresponding to the target promoter sequence. Inclusion of the whole-F2 primer in the second PCR is necessary to reduce non-specific amplification. This scheme was repeated for all *CPG* promoters that may have been introduced into the target line. Tetraploid transformants and those transformants harboring multiple constructs were not used for phenotypic analyses. As described in the ‘Results’ section, 95 transgenic crown violet lines possessing single constructs were generated via transformation with individual constructs (59 lines) or bulk constructs (36 lines); 52 transgenic crown violet lines were generated with a bulk plasmid of 8 constructs (*CpOMT*p and *CpSEP2*p constructs, inserts not determined); and 12 transgenic summerwave blue lines were generated with the *CpAP3B*p-*CpTCP1B-RD* construct.

### GUS staining

**Figure 2:**
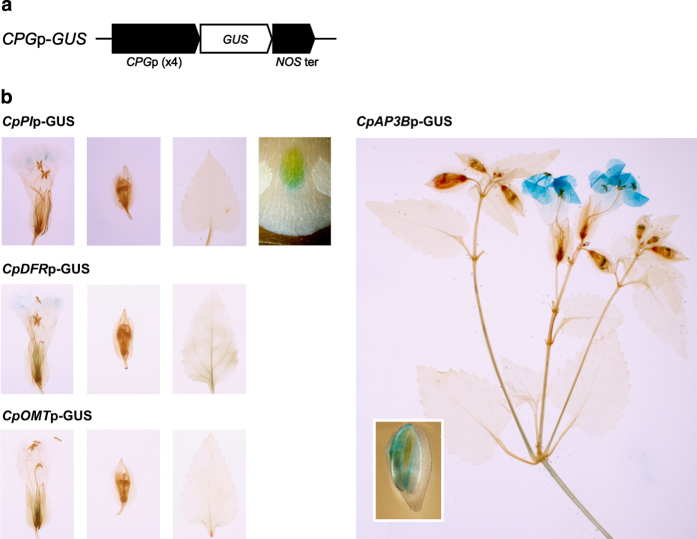
*CPG*p-*GUS* lines. (**a**) The genetic constructs introduced. Four cyclamen petal gene (*CPG*) promoters (*CpPI*p, *CpDFR*p, *CpOMT*p, and *CpAP3B*p) were used to drive *GUS* gene expression. ‘*NOS* ter’ represents the *NOPALINE SYNTHASE* terminator. (**b**) GUS staining of the *CpPI*p-*GUS* line (left to right: flower, floral bud, leaf, and blotch), the *CpDFR*p-*GUS* line (flower, floral bud and leaf), the *CpOMT*p-*GUS* line (flower, floral bud and leaf), and the *CpAP3B*p-*GUS* line (inset shows the anther excised from floral bud).

GUS staining was performed with T_1_ (first generation) transgenic plants (Figure 2). Plant tissues were pretreated with 90% acetone and placed under vacuum for 30 min in 100 mm sodium phosphate buffer (pH 7.0) containing 0.1% Triton X-100, 0.5 mg 5-bromo-4-chloro-3-indolyl-β-d-glucuronide (X-Gluc), 10 mm ethylenediaminetetraacetic acid (EDTA), 5 mm potassium ferricyanide and 5 mm potassium ferrocyanide. The tissues were then incubated in this solution at 37 °C in the dark overnight. The stained tissues were washed with 70% ethanol, treated with ethanol-acetic acid (6:1, v/v) overnight, washed again with 70% ethanol, and treated with chloral hydrate solution (chloral hydrate:glycerol:sterile water=8 g:1 mL:2 mL) overnight. Images were obtained using a single-lens reflex digital camera with a white backlight to observe whole staining or with a stereoscopic microscope (MZ16 FA, Leica, Wetzlar, Germany) to observe staining in small areas.

### Vascular structure

**Figure 1:**
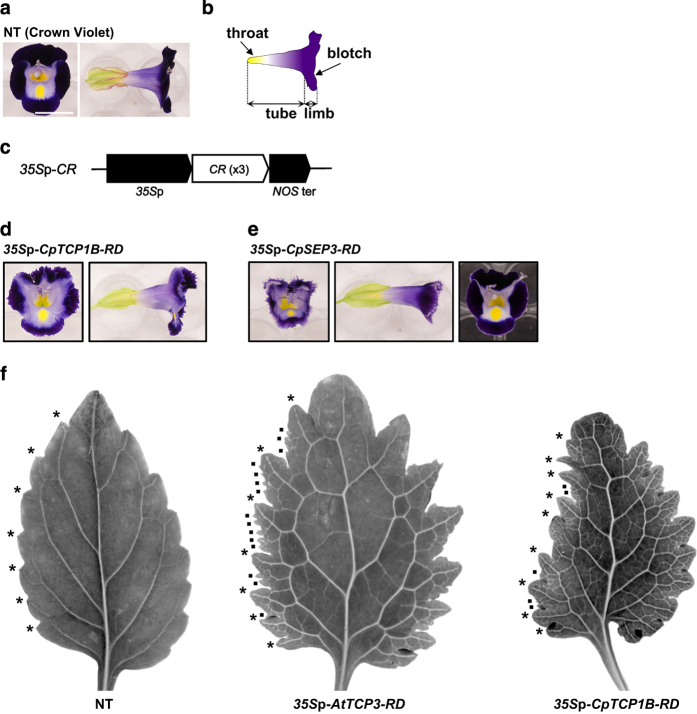
Structure of torenia flowers and the phenotypes of *35S*p-*CR* lines. (**a**) A non-transgenic (NT) Crown Violet flower. Bar=1 cm. (**b**) Schematic representation of the flower structure. (**c**) The genetic constructs introduced. *35S*p was combined with three chimeric repressor (*CR*) genes (*CpTCP1B-RD*, *CpSEP3-RD* or *AtTCP3-RD*). (**d**) A transgenic line expressing *35S*p-*CpTCP1B-RD*. (**e**) Two transgenic lines expressing *35S*p-*CpSEP3-RD*. (**f**) Leaf silhouette and vascular structure of the non-transgenic line, the *35S*p-*AtTCP3-RD* line, and the *35S*p-*CpTCP1B-RD* line. The primary serrations are indicated with asterisks, and the secondary serrations are indicated with dots only on the left side of the leaves.

**Figure 6:**
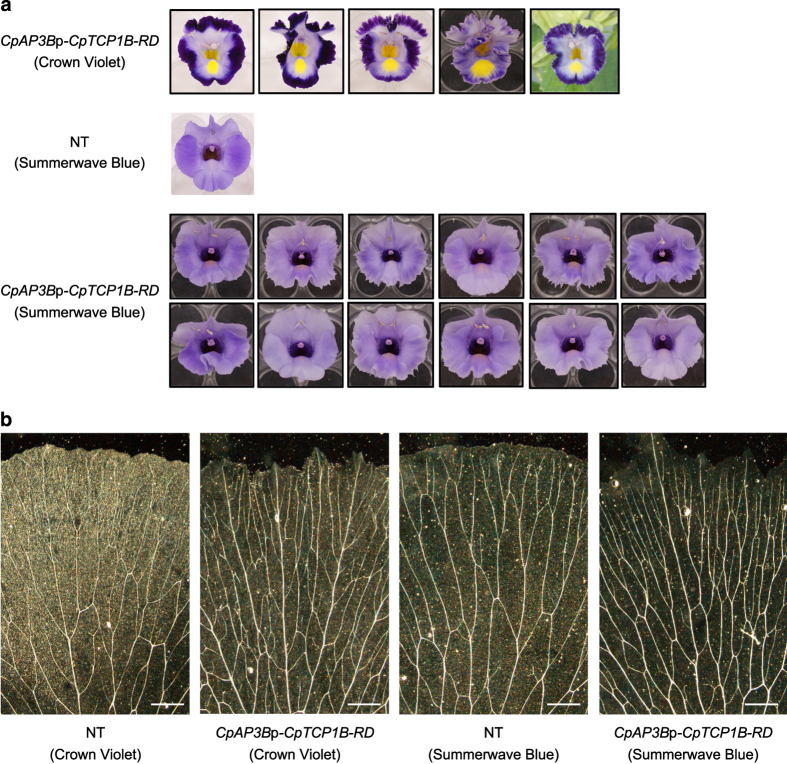
Phenotypes of *CpAP3B*p-*CpTCP1B-RD* lines. (**a**) Five *CpAP3B*p-*CpTCP1B-RD* lines of ‘Crown Violet,’ the non-transgenic cultivar ‘Summerwave Blue,’ and twelve *CpAP3B*p-*CpTCP1B-RD* lines of ‘Summerwave Blue.’ (**b**) Vascular structures in the petals of non-transgenic ‘Crown Violet,’ a *CpAP3B*p-*CpTCP1B-RD* line of ‘Crown Violet,’ non-transgenic ‘Summerwave Blue,’ and a *CpAP3B*p-*CpTCP1B-RD* line of ‘Summerwave Blue.’ Bars=1 mm.

Leaves were imaged using a single-lens reflex digital camera with a white backlight. The resulting digital images were retouched using Adobe Photoshop software (Adobe Systems Inc., San Jose, CA, USA) by converting the color image to a black and white image and then adjusting the brightness and contrast to make the vasculature clear (Figure 1f). Petals were treated with ethanol-acetic acid overnight and then placed in chloral hydrate solution overnight. The vasculature was observed under dark-field conditions with a stereoscopic microscope (MZ16 FA, Leica; Figure 6b).

### Petal cross-sections and scanning electron microscopy

**Figure 8:**
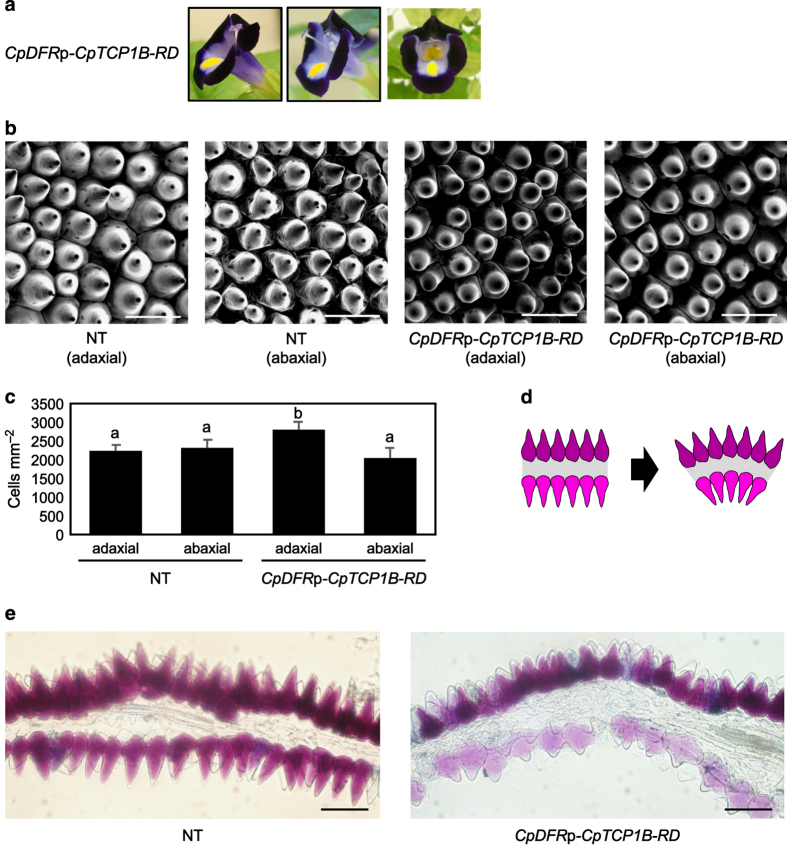
Microscopy analysis of *CpDFR*p-*CpTCP1B-RD* lines. (**a**) Phenotypes of three *CpDFR*p-*CpTCP1B-RD* lines. (**b**) Conical cells of the petals imaged via scanning electron microscopy on the adaxial/abaxial sides of the non-transgenic line and curled *CpDFR*p-*CpTCP1B-RD* line. (**c**) Conical cell density on petal surfaces. The data represent the mean and standard deviation (*n*=4). The values labeled with different letters (**a** or **b**) are significantly different from each other by Student’s *t*-test (*P*<0.05). (**d**) Schematic representation of the effect of differential cell densities. (**e**) Cross-section of the lobe of the non-transgenic line and curled *CpDFR*p-*CpTCP1B-RD* line. Bars=50 μm in all images.

To prepare petal cross-sections, agar with an isotonic solute was produced (2% agar, 500 mm trehalose, 5 mm CaCl_2_, 0.2% Tween-20). Side lobes excised from open flowers were sliced at a thickness of 55 μm with a vibrating microslicer (DTK-1000, Dosaka EM, Kyoto, Japan) and embedded in the agar. The slices were subsequently observed with a light microscope (AX70, Olympus, Tokyo, Japan; Figure 8e). The adaxial and abaxial surfaces of side lobes excised from open flowers were also observed with a scanning electron microscope (VE-7800, Keyence, Osaka, Japan; Figure 8b).

### Colorimetry

**Figure 5:**
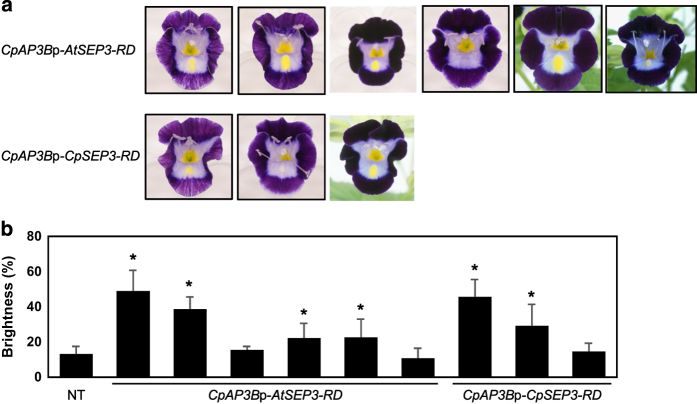
Comparison between the effects of *AtSEP3-RD* and *CpSEP3-RD*. (**a**) Phenotypes of six *CpAP3B*p-*AtSEP3-RD* lines and three *CpAP3B*p-*CpSEP3-RD* lines. (**b**) Brightness of the lobes measured in the non-transgenic line, *CpAP3B*p-*AtSEP3-RD* lines, and *CpAP3B*p-*CpSEP3-RD* lines. The data represent the mean and standard deviation (*n*=8). Asterisks indicate significant differences from the non-transgenic line by Student’s *t*-test (*P*<0.05). The data for transgenic lines correspond to flowers shown in (**a**) in the same order.

**Figure 7:**
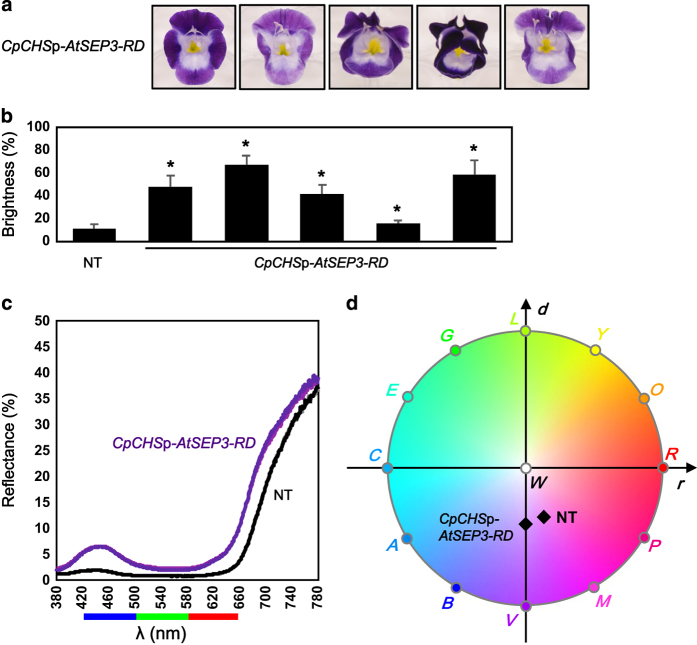
Colorimetric analysis of *CpCHS*p-*AtSEP3-RD* lines. (**a**) Phenotypes of five *CpCHS*p-*AtSEP3-RD* lines. (**b**) Brightness of the lobes measured in the non-transgenic line and *CpCHS*p-*AtSEP3-RD* lines. The data represent the mean and standard deviation (*n*=8). Asterisks indicate significant differences from the non-transgenic line by Student’s *t*-test (P<0.05). The data for the transgenic lines correspond to the flowers shown in (**a**) in the same order. (**c**) Reflectance spectra of visible light on the lobes of the non-transgenic line (black and gray lines) and of a light-colored *CpCHS*p-*AtSEP3-RD* line (violet and purple lines). The measurements were performed in two replications with different flowers; each replication produced similar results, and the lines (black and gray, or violet and purple) overlap with each other. The blue, green, and red bars at the bottom of the graph indicate the approximate color of light at each wavelength. (**d**) Colors (hue and saturation) of the non-transgenic line and a *CpCHS*p-*AtSEP3-RD* line plotted on a *round* color diagram.

The brightness of eight different parts of the same flowers was measured in images of flowers using Adobe Photoshop software (Figures 5b and 7b). The surface reflectance of the adaxial side of the side lobes was measured with an ultraviolet/visible (UV/VIS) spectrophotometer (UV-2450, Shimadzu, Kyoto, Japan; Figure 7c). RGB color under flat white light was calculated using a standardized CIE 1931 RGB color matching function. Hue, lightness, saturation, and coordinates in a round diagram were also calculated following previous reports (refs 23,24; Figure 7d). The recently developed round diagram is based on the RGB color system, which distributes hue angles and saturation values equally around a circle, consistent with our perception of color.^24^

## Results

### Isolation of cyclamen petal gene promoters

To express the chimeric repressors under the control of a variety of cyclamen promoters, we isolated 1.5-kb or longer upstream sequences (promoter sequences) of cyclamen petal genes (referred to as *CPG*s hereafter). Eleven homeotic MADS-box genes have been isolated from cyclamen, including *CpAP1*, *CpPI*, *CpAP3A*, *CpAP3B*, *CpSEP2*, *CpSEP3*, and *CpFLC*, which are all expressed in the petals (ref. 25; Table 1). In addition to these seven *CPG*s, we targeted three genes involved in anthocyanin pigmentation (*CpCHS*, *CpDFR* and *CpOMT*), which are also expressed in the petals (see footnote of Table 1). Here, the abbreviations *AP*, *PI*, *FLC*, *CHS*, *DFR* and *OMT* represent apetala, pistillata, flowering locus c, chalcone synthase, dihydroflavonol 4-reductase, and O-methyltransferase, respectively.

After adaptor PCR analysis, we successfully isolated the promoter sequences of 8 of the 10 target *CPG*s (*CpAP1*, *CpPI*, *CpAP3A*, *CpAP3B*, *CpSEP2*, *CpCHS*, *CpDFR*, and *CpOMT*). These promoter sequences are abbreviated as *CpAP1*p, *CpPI*p, *CpAP3A*p, *CpAP3B*p, *CpSEP2*p, *CpCHS*p, *CpDFR*p, and *CpOMT*p, respectively. All isolated promoter sequences (*CPG*p sequences), except for *CpAP3A*p (1179 bp), were longer than 1.5 kb, ranging from 1528 to 2030 bp (Table 1).

In this study, the transformed material was the torenia cultivar ‘Crown Violet’ (Figure 1a,b) unless otherwise indicated. Torenia presents flowers with compound petals (gamopetalous corolla). The basal part of the corolla (referred to as the ‘throat’ hereafter) is yellow, while the middle part is light purple. The basal and middle parts of the corolla are collectively referred to as the ‘tube.’ The top part of the corolla (the ‘limb’) is dark purple and is divided into four parts (‘lobes’). The lobe margins are rounded, and there is a yellow spot on the basal lobe of the corolla, which is referred to as the ‘blotch.’ The dark purple color of the limbs is caused by anthocyanin pigments.^26^

To examine promoter activity, we generated one transgenic torenia line expressing the *GUS* (β-glucuronidase) gene under four representative *CPG* promoters (*CpPI*p, *CpDFR*p, *CpOMT*p and *CpAP3B*p; Figure 2a). These transgenic plants were stained with a buffer containing 5-bromo-4-chloro-3-indolyl-β-D-glucuronide (X-Gluc), which is catabolized by the GUS enzyme to form a blue pigment. Blue pigmentation was observed only in the blotch in the *CpPI*p-*GUS* line (Figure 2b), indicating that *CpPI*p primarily drives gene expression in blotch cells. Weak staining was observed in the limbs of open flowers of the *CpDFR*p-*GUS* line (Figure 2b), while no staining was observed in the *CpOMT*p-*GUS* line (Figure 2b). The *CpAP3B*p-*GUS* line exhibited the greatest density of blue pigmentation among the four promoters examined (Figure 2b). Thus, strong staining was observed in the limbs of open flowers and in the anthers of floral buds, with weaker staining observed in the tubes of open flowers and in the basal part of the stem.

### Preparation of chimeric repressors

In this study, we used four chimeric repressors to modify torenia flowers. In addition to *AtTCP3-RD* and *AtSEP3-RD*, whose effects have been previously reported in torenia,^13,14^ a gene homologous to *AtSEP3* and a putative allele of *CpTCP1* from cyclamen were isolated, and corresponding chimeric repressors (*CpSEP3-RD* and *CpTCP1B-RD*) were prepared. Expression vectors in which *CpTCP1B-RD* or *CpSEP3-RD* was driven by the Cauliflower mosaic virus* *35S promoter (*35S*p) were used for functional characterization of these repressors, as was the expression vector of *AtTCP3-RD* for comparison (Figure 1c).

We generated one transgenic torenia line harboring the *35S*p-*CpTCP1B-RD* construct (Figure 1d) and two transgenic torenia lines harboring the *35S*p-*CpSEP3-RD* construct (Figure 1e). The line expressing *35S*p-*CpTCP1B-RD* possessed serrated petals. One of the lines expressing *35S*p-*CpSEP3-RD* exhibited jaggy petals, which were more pointed than the serrated petals. Compared with previous reports on *35S*p-*AtTCP3-RD* and *35S*p-*AtSEP3-RD*, *35S*p-*CpTCP1B-RD* showed similar effects to *35S*p-*AtTCP3-RD*, while *35S*p-*CpSEP3-RD* showed similar effects to *35S*p-*AtSEP3-RD* in transgenic torenia petals.

Torenia lines expressing *35S*p-*AtTCP3-RD* or *35S*p-*CpTCP1B-RD* produce petals and leaves with deeper and more complicated serrations. That is, non-transgenic leaves exhibit only ‘primary’ serrations, but the transgenic lines exhibit small ‘secondary’ serrations formed on the primary serrations as well as deeper primary serrations (Figure 1f). In our observations, we noted that the transgenic lines with more complicated serrations displayed more vascular branching in the leaves compared with the leaves of the non-transgenic line (Figure 1f). Vascular branching was particularly promoted at the leaf margins of these transgenic lines.

### Expression of chimeric repressors under the control of cyclamen petal gene promoters

**Figure 3:**
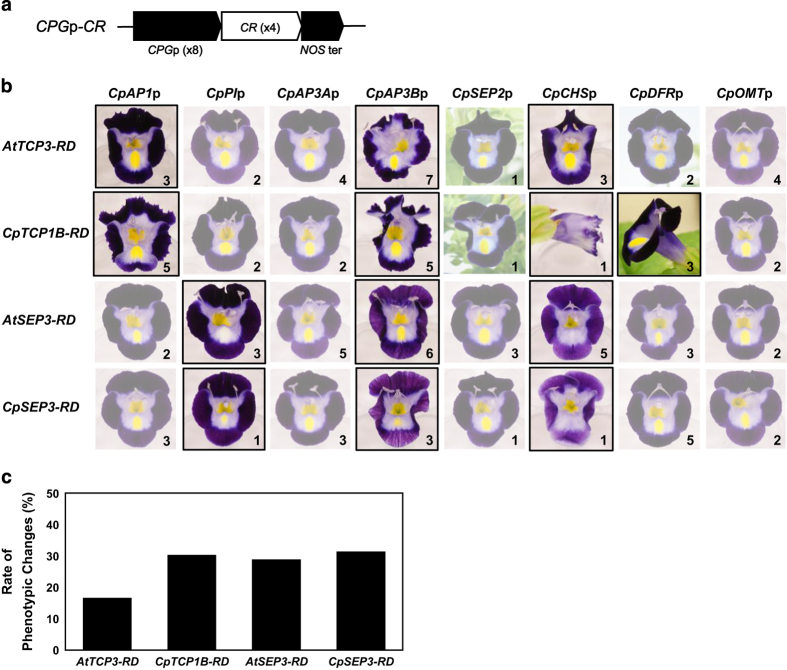
*CPG*p-*CR* lines. (**a**) The genetic constructs introduced. Eight *CPG* promoters (*CpAP1*p, *CpPI*p, *CpAP3A*p, *CpAP3B*p, *CpSEP2*p, *CpCHS*p, *CpDFR*p and *CpOMT*p) were combined with four chimeric repressors (*AtTCP3-RD*, *CpTCP1B-RD*, *AtSEP3-RD*, or *CpSEP3-RD*) in independent vectors. (**b**) Representative lines (those that were most clearly modified) transformed with each construct. The images are aligned by the introduced promoters and chimeric repressors. Phenotypically modified flowers are highlighted and surrounded with black lines. Images of phenotypically modified flowers are also surrounded with black lines in the other figures. The numbers at the bottom-right corner in each image indicate the numbers of transgenic lines generated. (**c**) The average rates of phenotypic changes for every chimeric repressor.

We prepared 32 different expression vectors using different combinations of the eight *CPG*p sequences and the four chimeric repressors (*CPG*p-*CR* vectors; Figure 3a). Torenia plants were transformed with each vector separately, and 59 lines were generated from these transformations. An additional 36 lines were generated through bulk transformation, whose T-DNA inserts were determined via PCR. Figure 3b shows representative lines with the most clearly modified flower phenotypes associated with each construct. Thirteen constructs resulted in phenotypes that differed from those of the non-transgenic line. *CpAP1*p generated flowers with jaggy margins when driving *AtTCP3-RD* or *CpTCP1B-RD*. *CpPI*p generated flowers without a blotch or with a smaller blotch (blotchless flowers) when driving *AtSEP3-RD* or *CpSEP3-RD*. *CpAP3B*p generated serrated and wavy flowers when driving *AtTCP3-RD* or *CpTCP1B-RD*. *CpAP3B*p also generated blotchless and striped flowers when driving *AtSEP3-RD* or *CpSEP3-RD*. *CpCHS*p generated ‘trumpet-shaped’ flowers with outer-curling limbs when driving *AtTCP3-RD* and ‘tube’ flowers without limbs when driving *CpTCP1B-RD*. *CpCHS*p generated blotchless and light-colored flowers when driving *AtSEP3-RD* or *CpSEP3-RD*. *CpDFR*p generated trumpet-shaped flowers when driving *CpTCP1B-RD*. *CpAP3A*p, *CpSEP2*p and *CpOMT*p combined with four repressors did not generate any phenotypic changes.

The absence of phenotypic changes associated with the constructs driven by *CpSEP2*p or *CpOMT*p was supported by the finding that a population of an additional 52 lines transformed with a bulk plasmid consisting of eight constructs driven by these promoters did not show any phenotypic changes (Supplementary Figure S1). Although it was not determined which construct was introduced into each line, eight constructs containing *CpSEP2p* or *CpOMTp* were expected to be randomly introduced into these lines. Excluding this population, one to seven transgenic lines per 32 constructs (approximately three lines, on average) were analyzed (Supplementary Figure S2). Approximately half of the lines were modified from the non-transgenic line when the 13 constructs were introduced, and the phenotypes were similar to each other when the transgenic lines possessed the same construct. It is possible that the remaining 19 constructs may produce phenotypic changes if larger populations of transgenic plants are generated. The average rate of phenotypically modified transgenic lines in the *CPG*p-*CR* population was ~30% for *CpTCP1B-RD*, *AtSEP3-RD* and *CpSEP3-RD*, while the rate for *AtTCP3-RD* was less than 20% (Figure 3c).

### Comparison between the chimeric repressors derived from Arabidopsis and cyclamen

*AtTCP3-RD* and *CpTCP1B-RD* caused similar phenotypic changes in transgenic torenia flowers, although both the degree and the rate of phenotypic changes appeared to be greater in association with *CpTCP1B-RD*. However, *AtSEP3-RD* and *CpSEP3-RD* caused similar phenotypic changes and showed a similar degree and rate of phenotypic changes (Figures 3b and Supplementary Figure S2). These trends were reconfirmed through an analysis of representative constructs.

**Figure 4:**
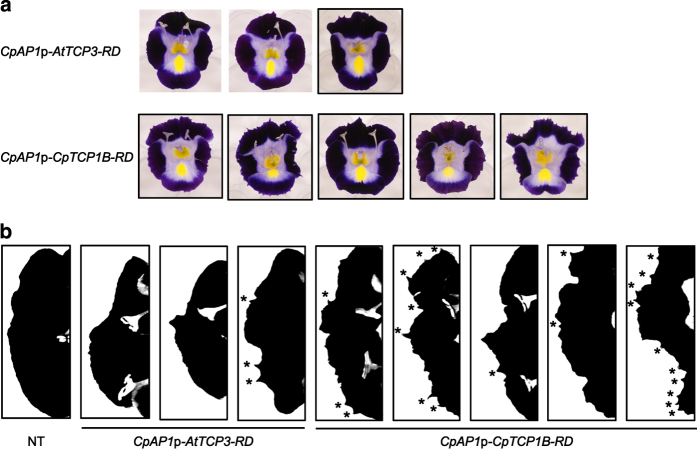
Comparison between the effects of *AtTCP3-RD* and *CpTCP1B-RD*. (**a**) Phenotypes of three *CpAP1*p-*AtTCP3-RD* lines and five *CpAP1*p-*CpTCP1B-RD* lines. (**b**) Silhouettes of the top lobes of the non-transgenic line, *CpAP1*p-*AtTCP3-RD* lines, and *CpAP1*p-*CpTCP1B-RD* lines. Serrations are indicated by asterisks. The silhouettes of the transgenic lines correspond to the flowers shown in (**a**) in the same order.

The *CpAP1*p-*AtTCP3-RD* and *CpAP1*p-*CpTCP1B-RD* constructs were chosen for comparison of the effects of *AtTCP3-RD* and *CpTCP1B-RD*. Both constructs generated serrations at the lobe margins, but the degree of serration tended to be greater in *CpAP1*p-*CpTCP1B-RD* lines (Figure 4). The *CpAP3B*p-*AtSEP3-RD* and *CpAP3B*p-*CpSEP3-RD* constructs were chosen for a comparison of the effects of *AtSEP3-RD* and *CpSEP3-RD*. Both constructs decreased the purple coloration of the lobes and the yellow coloration of the blotch (Figure 5a). The degree of phenotypic changes was quantified based on the brightness of the lobes (Figure 5b); when the purple coloration is reduced by the chimeric repressors to a greater extent, the lobes become brighter. The distribution of lobe brightness in the transgenic lines was similar between *AtSEP3-RD* and *CpSEP3-RD*, suggesting that *AtSEP3-RD* and *CpSEP3-RD* have similar effects in torenia.

### Microscopic analysis of serrated flowers

Although the beauty of flowers will always be subjective depending on personal preferences, the flowers among these lines showing the greatest degree of modification are the serrated, wavy (and light-colored) flowers associated with *CpAP3B*p-*CpTCP1B-RD*, the blotchless, light-colored flowers associated with *CpCHS*p-*AtSEP3-RD*, and the trumpet-shaped flowers associated with *CpDFR*p-*CpTCP1B-RD*. In addition, all flowers on individual plants showed similar, consistent phenotypes. Examples of plants producing these three types of flowers are shown in Supplementary Figure S3. The flowers generated by these three constructs were further analyzed.

Phenotypic changes were observed to varying degrees in all five *CpAP3B*p-*CpTCP1B-RD* lines of the ‘Crown Violet’ cultivar (Figure 6a). We also generated 12 *CpAP3B*p-*CpTCP1B-RD* lines of the torenia cultivar ‘Summerwave Blue,’ all of which exhibited serrations and/or slight reductions in purple coloration (Figure 6a). We observed vascular structures on the serrated side lobes of *CpAP3B*p-*CpTCP1B-RD* lines (Figure 6b). Vascular branching was enhanced by the introduction of the *CpAP3B*p-*CpTCP1B-RD* construct in both the crown violet and summerwave blue genetic backgrounds.

### Colorimetric analysis of light-colored flowers

Transgenic lines harboring the *CpCHS*p-*AtSEP3-RD* construct produced blotchless and light-colored flowers (Figure 7a). The lobe colors of all five lines were brighter than those of the non-transgenic line (Figure 7b). Although it may not be clear from the digital images (due to a problem with color regeneration from the digital camera), the light-colored transgenic flowers tended to be slightly more bluish than the non-transgenic flowers. To examine this phenomenon more precisely, we analyzed the side lobes of one of the *CpCHS*p-*AtSEP3-RD* lines following rigorous colorimetric procedures.

First, we measured the reflectance spectra of visible wavelengths on the lobes with a spectrophotometer (Figure 7c). Wavelengths in the ranges of 420–500, 500–580 and 580–660 nm roughly correspond to blue, green and red coloration, respectively.^24^ The reflectance recorded in the non-transgenic line consisted of a medium peak in the blue range (~440 nm), low values in the green range, and a large slope in the red range. Reflectance was enhanced for all wavelengths in the *CpCHS*p-*AtSEP3-RD* line, but the positions of the blue peak and the red slope were similar to those in the non-transgenic line.

We transformed the visible light spectra of the reflectance of the lobes to RGB color using an ‘RGB color matching function.’ In general, color is composed of three factors: hue (for example, red, yellow, green and purple), lightness (brightness), and saturation (vividness). In colorimetry, hue is expressed as the hue angle (0–360°), while lightness and saturation are expressed as ratios (0–100%). The non-transgenic line and the *CpCHS*p-*AtSEP3-RD* line exhibited hue angles (H^RGB^ value) of 290° and 269°, lightness values (L^RGB^) of 4% and 12%, and saturation values (S^RGB2^) of 37% and 40%, respectively. The lobe colors were then plotted on a ‘*round* diagram’ (Figure 7d).

### Microscopic observation of trumpet-shaped flowers

Trumpet-shaped flowers were generated in two of the three *CpDFR*p-*CpTCP1B-RD* lines (Figure 8a). We examined the effect of this construct on the generation of trumpet-shaped flowers by performing microscopic observations of the side lobes of both the non-transgenic line and a trumpet-shaped *CpDFR*p-*CpTCP1B-RD* line.

We directly observed the density of conical cells on the adaxial (upper) and abaxial (lower) surfaces of the side lobes in the non-transgenic line and the *CpDFR*p-*CpTCP1B-RD* line through scanning electron microscopy (Figure 8b). The conical cell density was significantly greater on the adaxial surface than on the abaxial surface of the *CpDFR*p-*CpTCP1B-RD* line (Figure 8c). Figure 8d illustrates a possible geometric effect of increased conical cell density on the adaxial surface of the lobes, while Figure 8e shows cross-sections of fresh lobes prepared from both the non-transgenic and *CpDFR*p-*CpTCP1B-RD* lines.

## Discussion

### Activities of *CPG* promoters in torenia and their relationship with flower phenotypes

**Figure 9:**
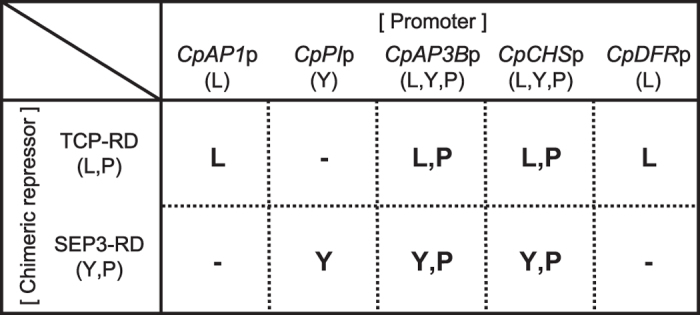
Relationship between *CPG* promoters, chimeric repressors, and the categories of flower phenotypes. The phenotypes of transgenic torenia flowers generated by a combination of five *CPG* promoters (*CpAP1*p, *CpPI*p, *CpAP3B*p, *CpCHS*p or *CpDFR*p) and two types of chimeric repressors (*TCP-RD* or *SEP3-RD*) were categorized as ‘L’ (lobe morphology), ‘Y’ (yellow color of the blotch), or ‘P’ (purple color of the petal). The potential effects of the *CPG* promoters and chimeric repressors, as deduced from the flower phenotypes, are also indicated in parentheses.

The sizes of the isolated promoter sequences (all longer than 1.5 kb, except for *CpAP3A*p) are expected to be sufficient for the expression of downstream genes because conserved domains are found within 1.0 kb of promoter sequences for dicot MADS-box genes.^27^ In addition, floral gene promoters ranging from 1394 bp to 2083 bp derived from Arabidopsis or torenia have been successfully used previously for gene expression in torenia petals.^16^ Different combinations of *CPG* promoters and chimeric repressors generated different types of flowers in torenia, although some specific combinations did not result in any phenotypic changes (Figure 3). The observed phenotypic changes in the petals fell into three categories: changes in lobe morphology (e.g., serrated lobes), changes in the yellow coloration of the blotch, and changes in the purple coloration of the limbs. Figure 9 shows the phenotypic changes in these categories caused by different combinations of *CPG* promoters and chimeric repressors (*TCP-RD* or *SEP3-RD*). For example, because transgenic lines harboring *CpPI*p-*SEP3-RD* constructs exhibited a blotchless phenotype, they were considered modified in category ‘Y’ (yellow) but not in categories ‘L’ (lobes) and ‘P’ (purple). In contrast, transgenic lines harboring *CpAP3B*p-*TCP-RD* constructs were modified in categories ‘L’ and ‘P,’ but these lines always exhibited normal blotch colors.

This categorization of the observed phenotypic changes in the transgenic lines allowed us to deduce the capacity of *CPG* promoters and chimeric repressors to modify these respective categories of flower phenotypes. Thus, *TCP* repressors can modify L-type and P-type phenotypes, while *SEP3* repressors can modify Y-type and P-type phenotypes. *CpAP1*p and *CpDFR*p can modify L-type phenotypes; *CpPI*p can modify Y-type phenotypes; and *CpAP3*Bp and *CpCHS*p can modify all three categories. Each phenotype category is modified only when both the promoter and repressor have the ability to modify it. This model, based on the differential activities of both *CPG* promoters and chimeric repressors, would reasonably explain the variable flower phenotypes generated by different constructs.

The tissue-specific activities of the four promoters (Figure 2) are also consistent with this model. *CpPI*p activity was specifically detected in the blotch, which explains why *CpPI*p can modify only the Y-type phenotype and why the flower throat remained yellow in the lines possessing the *CpPI*p-*SEP3-RD* construct. In contrast, *CpAP3B*p activity was observed in the whole petal, explaining why this promoter can modify all three phenotypic categories. *CpDFR*p was only weakly active in the limbs, and this promoter specifically modified L-type phenotypes. L-type phenotypes may be modified by TCP repressors more easily compared with P-type phenotypes. No activity of *CpOMT*p was detected in our experiments, and this promoter accordingly did not cause any changes in flower phenotypes. As *CpAP3A*p or *CpSEP2*p also did not cause phenotypic changes, these two promoters may not be active in torenia flowers.

The observed activity of *CpAP3B*p in the petals and stamens is in accord with its phylogenetic classification as a class-B MADS-box gene, although no phenotypic changes were observed in the stamens in this study. The blotch-specific activity of *CpPI*p was unexpected, considering its classification as a class-B *MADS-box* gene, and the GUS staining associated with *CpDFR*p in this study appeared weaker than the GUS staining associated with the torenia *DFR* promoter in a previous study.^16^ Torenia may be lacking in *trans* regulators that properly recognize and strongly drive the transgenic cis regulators *CpPI*p and *CpDFR*p. Due to their specific expression patterns, the activities of these promoters may result in a lack of phenotypic changes in transgenic torenia in some cases and unexpected flower phenotypes in others.

### Role of TCP transcription factors in the regulation of cell proliferation

TCP transcription factors are known to regulate cell proliferation.^28^ Among the *TCP* family genes, *AtTCP3* and *CpTCP1* are close homologs of the *CINCINNATA* gene (*AmCIN*) of snapdragon (*Antirrhinum majus*; ref. 15). A mutation in the *AmCIN* gene causes crinkly (curly) leaves, particularly at the margins, and arrests the growth of the lobes in snapdragon,^29^ while a snapdragon mutant harboring mutations in the *TCP* genes *DICHOTOMA* and *CYCLOIDEA* exhibits a rounded corolla.^30^ Thus, TCP has various effects on flower morphogenesis.

The curly lobes observed in the *CpDFR*p-*CpTCP1B-RD* line (Figure 8a) were consistent with the mutant phenotype of the *AmCIN* gene in snapdragon. Outer curling of the lobes (that is, epinasty) is caused by an imbalance in cell proliferation and/or cell expansion between the adaxial (inner) and abaxial (outer) surfaces of the lobes. Microscopic analysis revealed an increased density of conical cells only on the adaxial surface of the *CpDFR*p-*CpTCP1B-RD* line (Figure 8c), indicating that *CpTCP1B-RD* accelerated cell proliferation on the adaxial surface of transgenic plants. The conical cells of the *CpDFR*p-*CpTCP1B-RD* line also tended to be flatter than those of the non-transgenic line (Figure 8e), similar to what was observed in a snapdragon *AmCIN* gene mutant.^31^

In addition to the petal curvature caused by *CpDFR*p-*CpTCP1B-RD*, the *TCP* repressors also generated complicated serrations in leaves and petals (Figures 1 and 6). The effects of TCP repressors on serrations can be divided into two categories. First, TCP repressors cause deeper primary serrations. This effect is consistent with the CUC2 (CUp-shaped Cotyledon 2)-mediated formation and regulation of leaf serrations.^32^ Indeed, *CUC2* expression is elevated in Arabidopsis plants with mutations in TCP genes.^33^ Second, TCP repressors generate small secondary serrations in leaves (Figure 1f) and possibly in petals (Figure 6b). The positions of the secondary serrations appear to overlap with the positions at which additional vasculature is formed in transgenic lines. This positive and possibly simultaneous regulation of the formation of secondary serrations and vascular branching should be independent of the CUC2 pathway because CUC2 is known to neither induce secondary serrations nor regulate vascular branching.^32^

A mutation in the *FRILL1* gene also causes frilled flowers in Arabidopsis,^34^ and the vascular pattern is altered in such flowers.^35^ The frilled phenotype of this mutant is attributed to the differential regulation of endoreduplication,^36^ a phenomenon in which some diploid (2C) cells are converted to tetraploid (4C), octaploid (8C) or hexadecaploid (16C) cells with an enlarged cell size. Cell ploidy analyses have shown that endoreduplication frequently occurs in vegetative cells of Arabidopsis (a diploid) (for example see refs. 37,38). However, there is no sign of endoreduplication in diploid torenia leaves; i.e., there is only a single peak corresponding to diploid (2C) cells (Kasajima et al., unpublished). Although we do not have sufficient data to reach a definite conclusion, vascular branching, rather than endoreduplication, may be linked to the serrations observed in torenia.

Phenotypic changes caused by SEP3 repressors are not necessarily strong, but the reduction of purple coloration may be a sign of the conversion of petals to sepals. This possibility could be examined using gene expression analysis in future studies.

### Why do transgenic plants with *CpCHS*p-*SEP3-RD* constructs produce bluish flowers?

*CpCHS*p-*SEP3-RD* constructs generated blotchless, light-colored flowers (Figure 3). The characteristics of these flowers lie partly in the simple balance of colors, in the absence of a yellow blotch, and in lighter lobe colors. The lobe coloration of these lines also appeared somewhat bluish when directly observed with the naked eye, which was confirmed by colorimetric analysis (Figure 7). Judging from the obtained colorimetric values, the non-transgenic flowers were magenta (red-purple), while the *CpCHS*p-*AtSEP3-RD* flowers were violet (blue-purple) and three times lighter. The purple petal color of torenia is caused by anthocyanins.^26^

The color of purple flowers is affected by changes in the spectra of incident light. Therefore, the abovementioned flower colors (magenta and violet) were calculated under a hypothetical ‘flat’ white light, which mimics the solar spectrum, with an even distribution of spectral power across all visible wavelengths (380–780 nm). The color of purple flowers, including that of purple torenia flowers, becomes bluish under white light-emitting diode (LED) light and reddish under incandescent light due to the colorimetric phenomenon known as the ‘alexandrite effect’ or ‘color change’.^24^ This may be the principal reason that the color (hue) of a given purple flower appears different between images.

The observed difference in the hues between the non-transgenic line and the *CpCHS*p-*AtSEP3-RD* line may have been caused by a colorimetric phenomenon other than changes in the chemical structure of anthocyanins. In a previous report, a bluish torenia line (cultivar ‘Crown Violet’) was generated by suppressing the *DFR* gene.^26,39^ Suppression of the *DFR* gene modified co-pigment accumulation and shifted the spectral pattern of light absorbance to the right (that is, to longer wavelengths). However, there was no obvious shift or changes in the shape of the reflectance spectrum in the *CpCHS*p-*AtSEP3-RD* line (Figure 7c), except that the entire spectrum was magnified to approximately three times that of the non-transgenic line. Although we did not measure anthocyanin concentrations in this study, we deduced that ‘dichromatism’ causes the hue to change in the presence of different concentrations of purple anthocyanins. Strong red reflectance will cause the flower color to be reddish at high anthocyanin concentrations in the non-transgenic line, whereas intermediate blue reflectance will be enhanced in the presence of lower concentrations of purple anthocyanins in the *CpCHS*p-*AtSEP3-RD* line, causing the flower to become bluish. The exponential relationship between light transmittance and pigment concentrations, as described by the Beer-Lambert law, causes this kind of alteration in the balance between the intensities of different wavelengths at different pigment concentrations.^23^ The concentration and chemical structure of anthocyanins in the petals of *CpCHS*p-*AtSEP3-RD* lines should be examined in the future to obtain further insight into the color of these flowers.

## Conclusion

In this study, we newly isolated 8 promoters of genes expressed in cyclamen petals and prepared 32 constructs. We then identified 13 genetic constructs that modify flower phenotypes, including *CpCHS*p-*AtSEP3-RD*, *CpDFR*p-*CpTCP1B-RD*, and *CpAP3B*p-*CpTCP1B-RD*, by ‘screening’ the constructs in the model flower torenia. These three constructs generated pale bluish petals, trumpet-like petals, and serrated petals, respectively. We also examined the cellular effects underlying these phenotypic changes and deduced that the changes are linked to dichromatism, imbalanced cell proliferation, and excessive vascular branching, respectively. Taken together, the information and genetic resources generated in this study will benefit future molecular flower breeding efforts and further characterization of these valuable transgenic plants.

## Figures and Tables

**Table 1:** Properties of cyclamen petal genes (*CPG*s) and their promoters.

*Gene*	*MADS class*	*INSDC*^a^ *accession number*		*Promoter length*^b^
		*Gene*	*Promoter*	*(bp)*
*CpAP1*	A	AB600230.1	LC176494	1596
*CpPI*	B	AB600234.2	LC176500	2030
*CpAP3A*	B	AB600231.1	LC176495	1179
*CpAP3B*	B	AB600232.1	LC176496	1554
*CpSEP2* (*CpMADS1*)^c^	E	AB600235.1	LC176501	1863
*CpSEP3* (*CpMADS2*)^c^	E	AB600236.1	—	—
*CpFLC* (*CpMADS3*)^c^	—	AB600233	—	—
*CpCHS*^d^	—	DD354767	LC176497	1555
*CpDFR*^d^	—	DD354768	LC176498	1528
*CpOMT*^d^	—	DD248507	LC176499	1655

a International Nucleotide Sequence Database Collaboration (collaboration between DDBJ, EMBL-EBI, and NCBI; http://www.insdc.org/).

b Lengths of the isolated sequences, upstream of the start codon (ATG).

c The original names for *CpSEP2*, *CpSEP3*, and *CpFLC* were *CpMADS1*, *CpMADS2* and *CpMADS3*, respectively.^25^

d These genes are expressed in cyclamen petals, as their cDNA sequences were isolated from cyclamen petals (Japan patents 2006-115861 and 2005-312388; https://www.j-platpat.inpit.go.jp/web/tokujitsu/tkbs/TKBS_GM101_Top.action, Japanese).
