# Supplementary figures and images for: Combination of Cyclamen persicum Mill. floral gene promoters and chimeric repressors for the modification of ornamental traits in Torenia fournieri Lind
Source: Hortic Res. 2017 Mar 22;4:17008–. doi: 10.1038/hortres.2017.8 (PMC5386234; doi:10.1038/hortres.2017.8)

## Slide 1
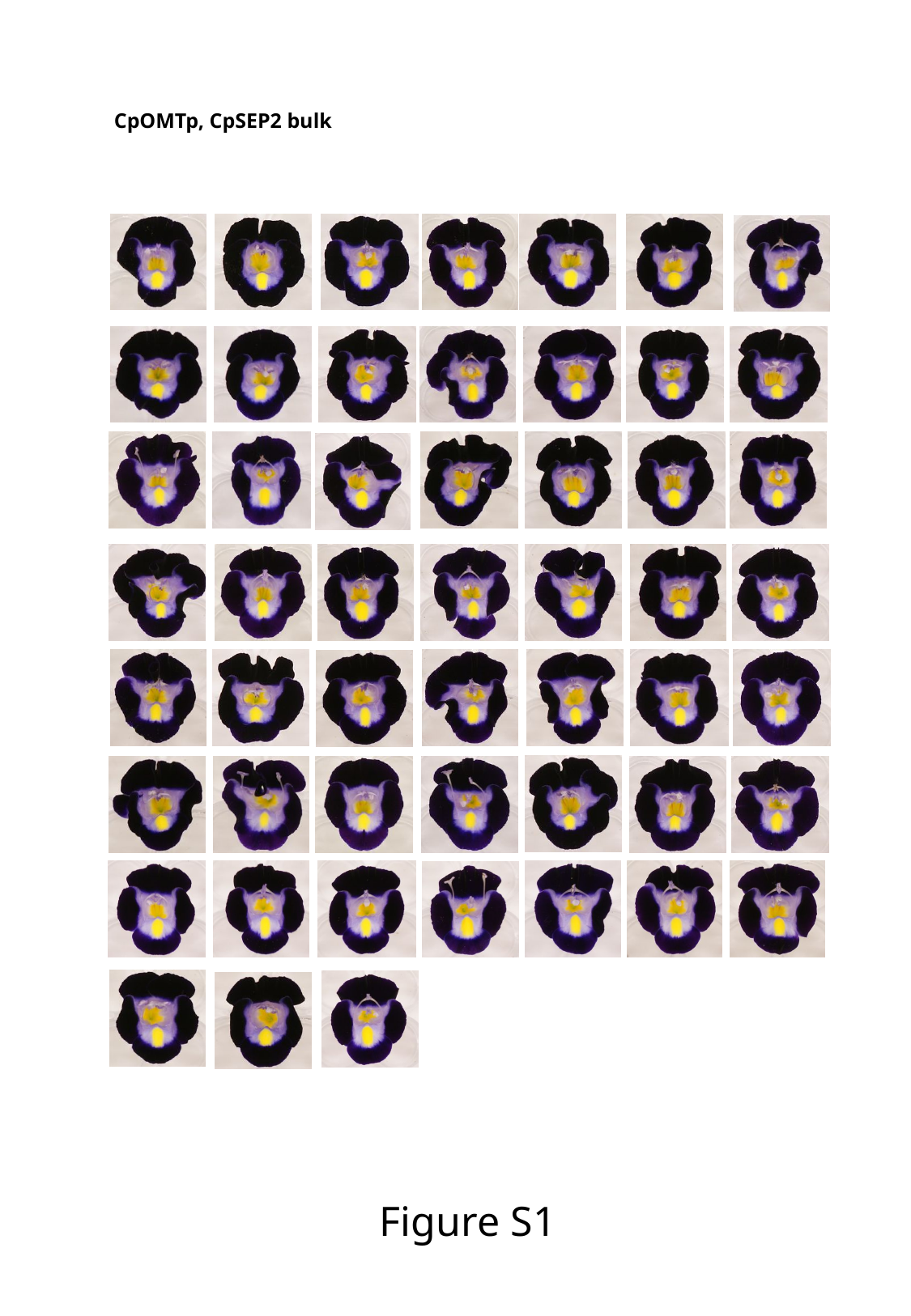

CpOMTp, CpSEP2 bulk
Figure S1

Supplement: Supplementary Figure 1 [file hortres20178-s1.ppt]

## Slide 1
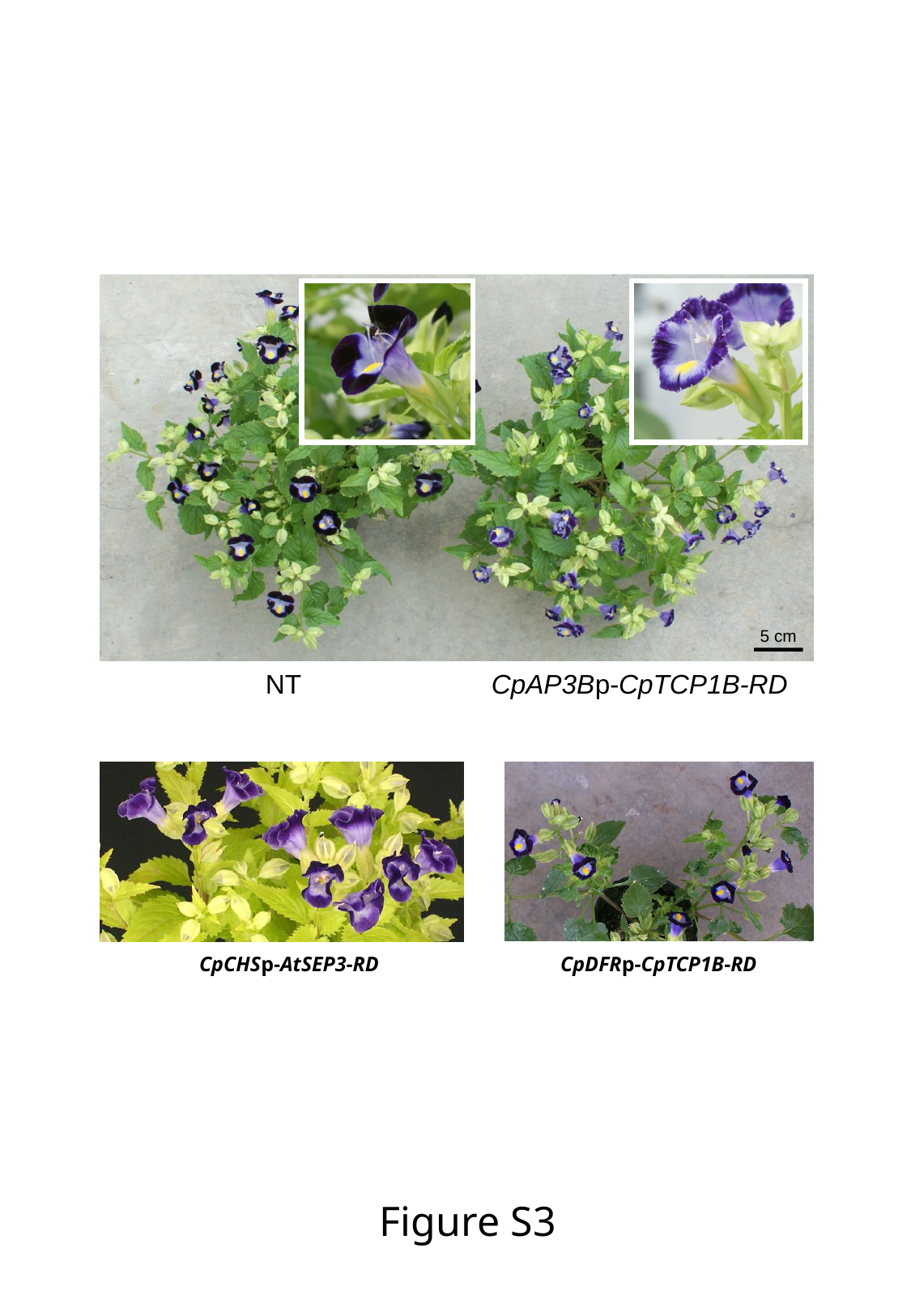

5 cm
NT
CpAP3Bp-CpTCP1B-RD
CpCHSp-AtSEP3-RD
CpDFRp-CpTCP1B-RD
Figure S3

Supplement: Supplementary Figure 3 [file hortres20178-s3.ppt]
