# Supplementary material for: Combination of Cyclamen persicum Mill. floral gene promoters and chimeric repressors for the modification of ornamental traits in Torenia fournieri Lind
Source: Hortic Res. 2017 Mar 22;4:17008–. doi: 10.1038/hortres.2017.8 (PMC5386234; doi:10.1038/hortres.2017.8)
Supplement: Supplementary Figure 2 [file hortres20178-s2.ppt]

## Slide 1
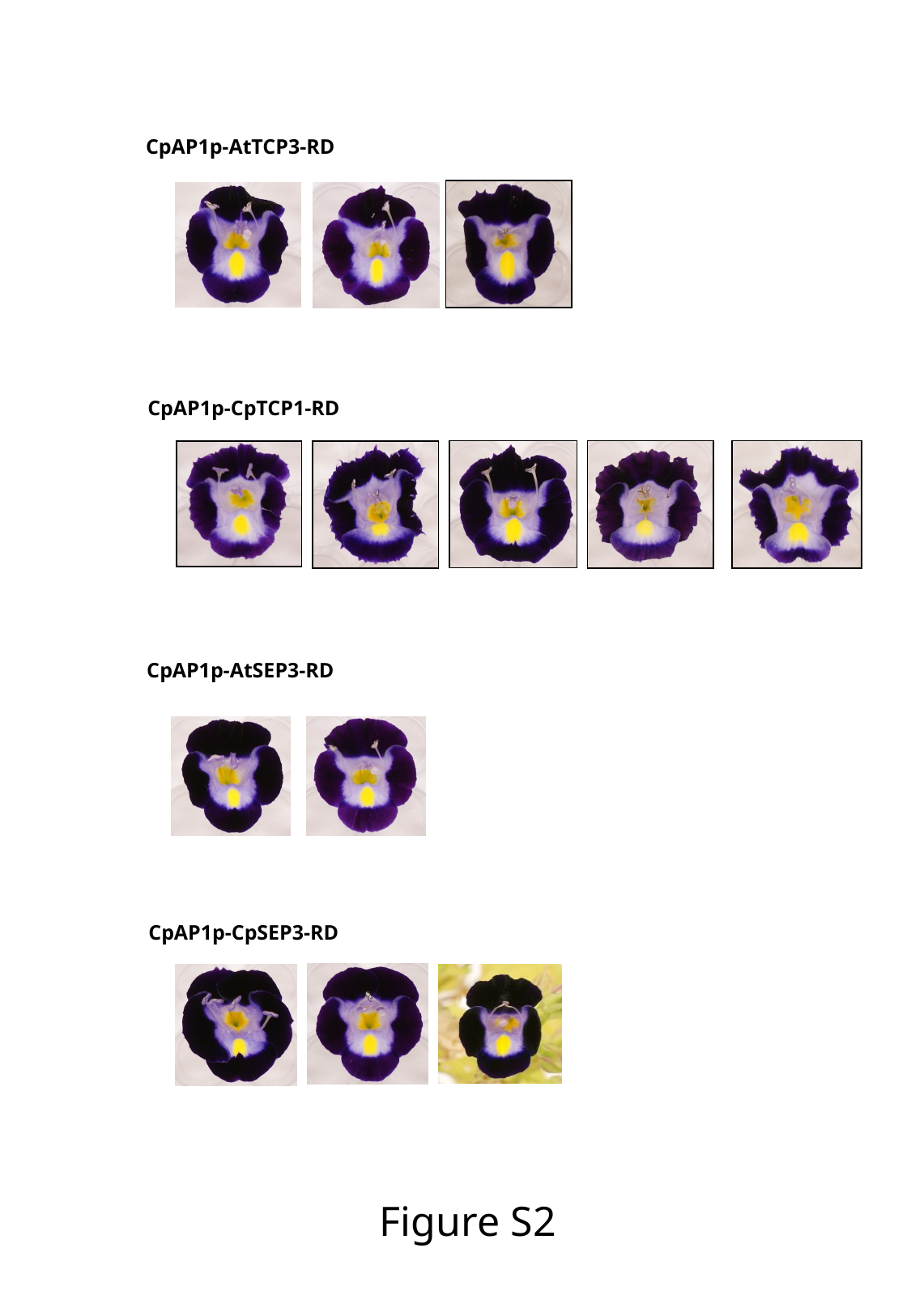

CpAP1p-AtTCP3-RD
CpAP1p-CpTCP1-RD
CpAP1p-AtSEP3-RD
CpAP1p-CpSEP3-RD
Figure S2

## Slide 2
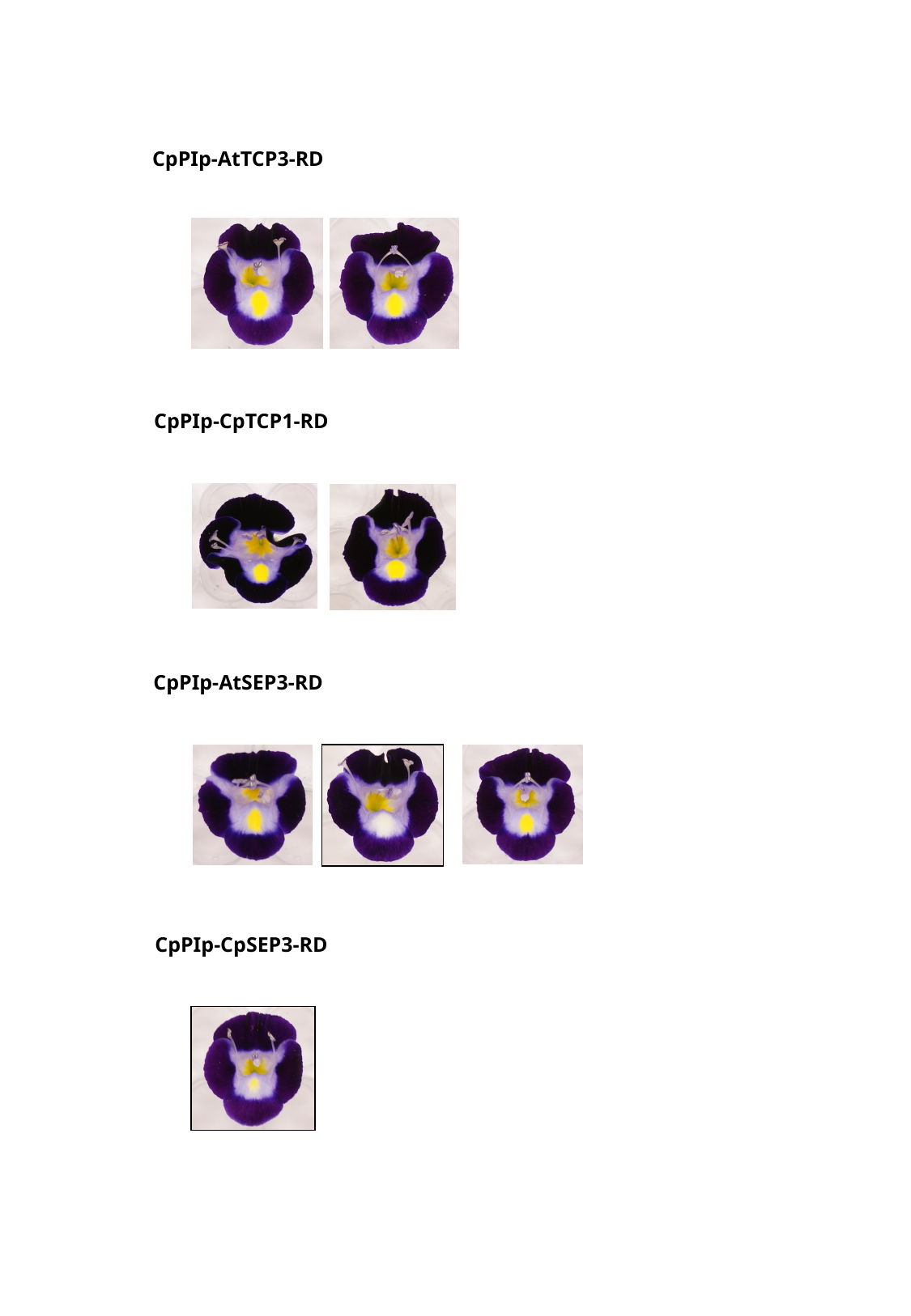

CpPIp-AtTCP3-RD
CpPIp-CpTCP1-RD
CpPIp-AtSEP3-RD
CpPIp-CpSEP3-RD

## Slide 3
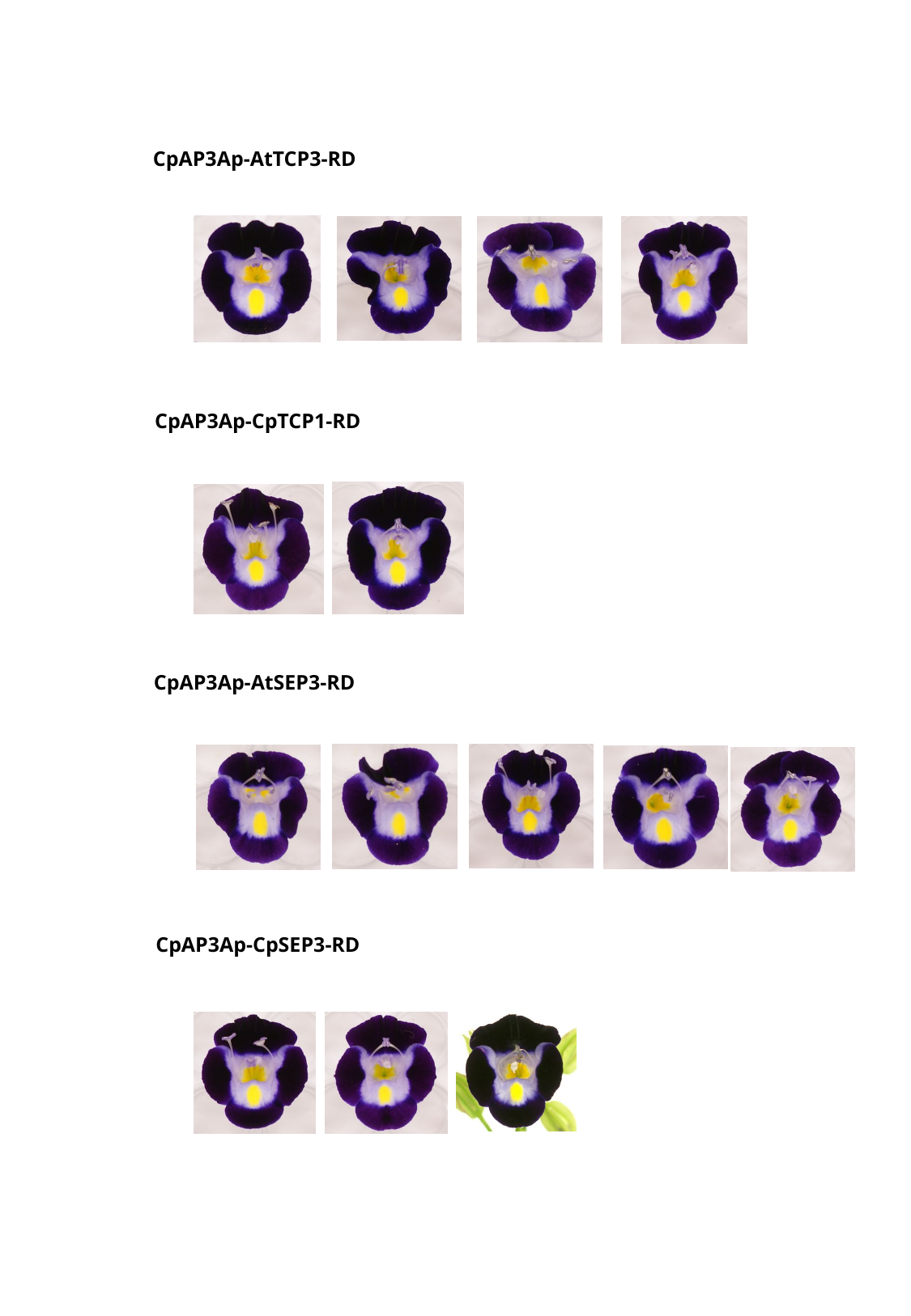

CpAP3Ap-AtTCP3-RD
CpAP3Ap-CpTCP1-RD
CpAP3Ap-AtSEP3-RD
CpAP3Ap-CpSEP3-RD

## Slide 4
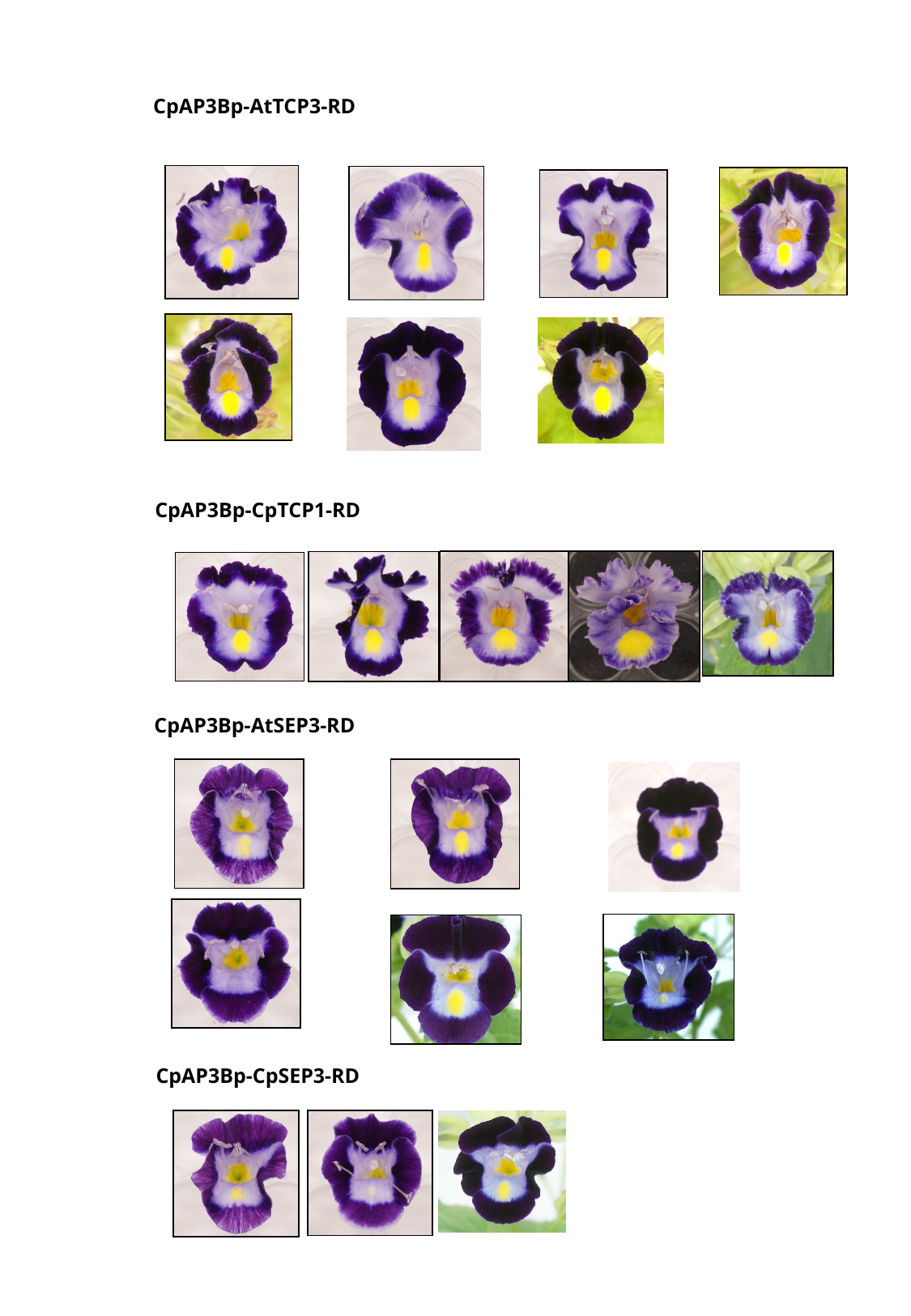

CpAP3Bp-AtTCP3-RD
CpAP3Bp-CpTCP1-RD
CpAP3Bp-AtSEP3-RD
CpAP3Bp-CpSEP3-RD

## Slide 5
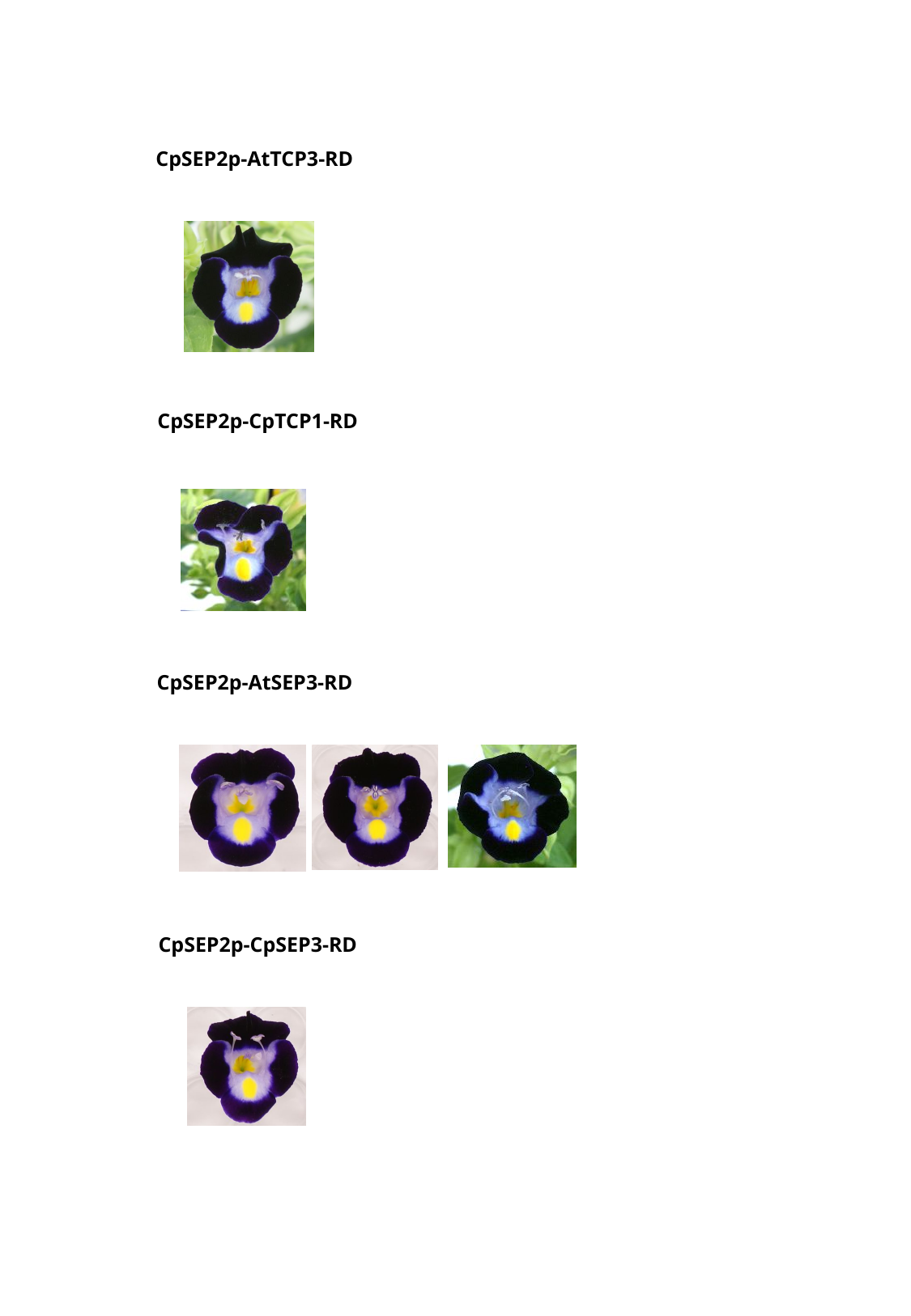

CpSEP2p-AtTCP3-RD
CpSEP2p-CpTCP1-RD
CpSEP2p-AtSEP3-RD
CpSEP2p-CpSEP3-RD

## Slide 6
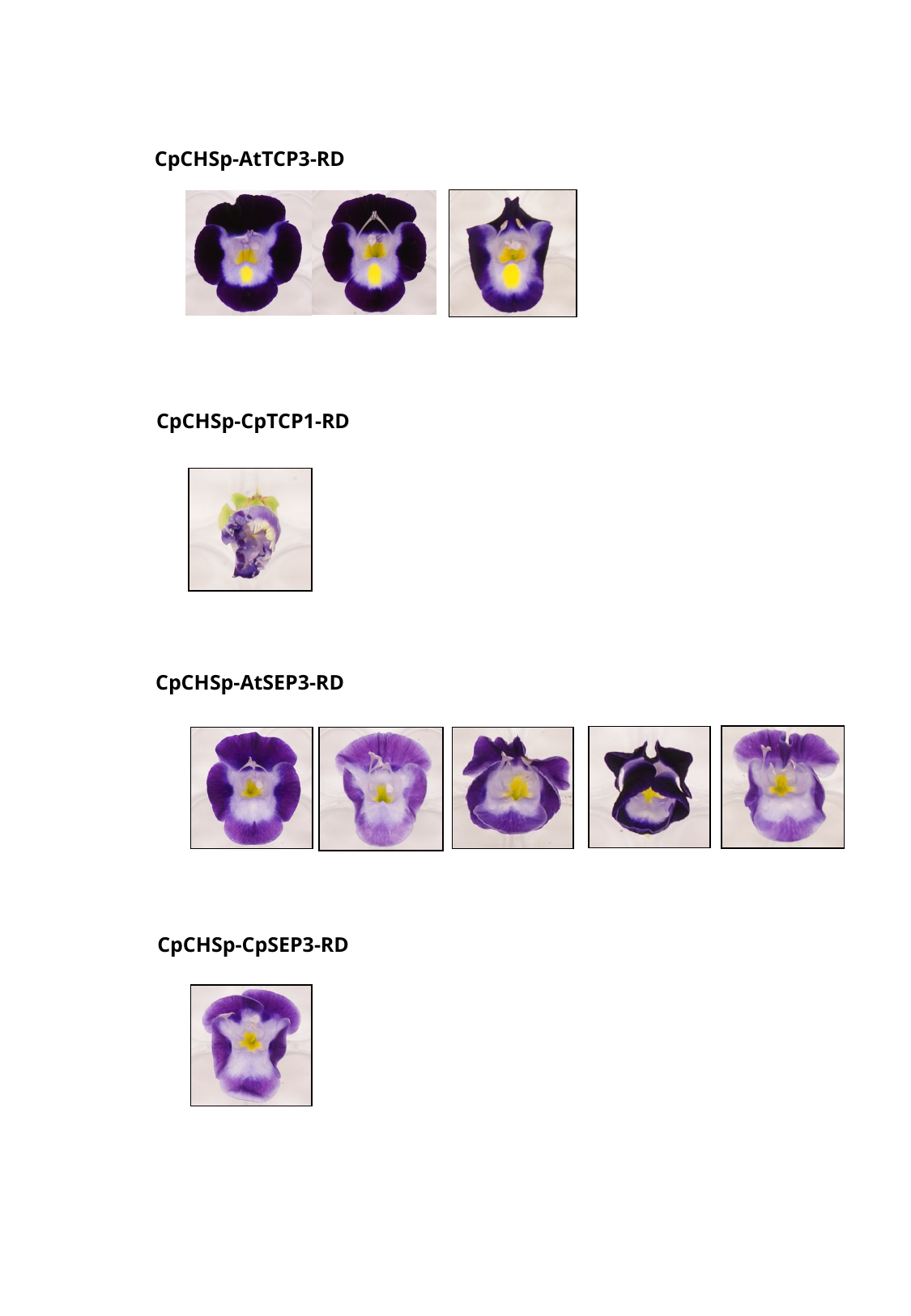

CpCHSp-AtTCP3-RD
CpCHSp-CpTCP1-RD
CpCHSp-AtSEP3-RD
CpCHSp-CpSEP3-RD

## Slide 7
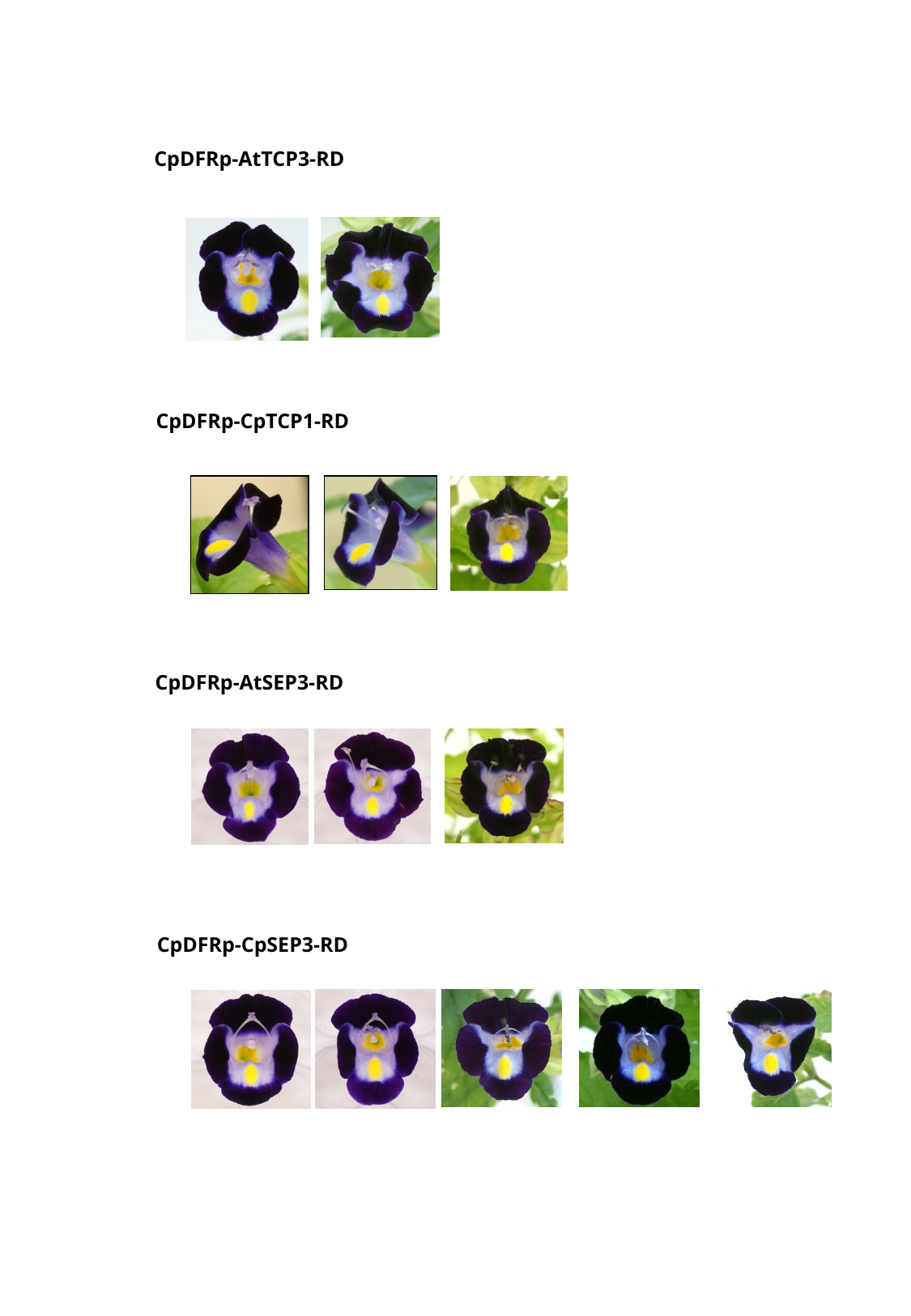

CpDFRp-AtTCP3-RD
CpDFRp-CpTCP1-RD
CpDFRp-AtSEP3-RD
CpDFRp-CpSEP3-RD

## Slide 8
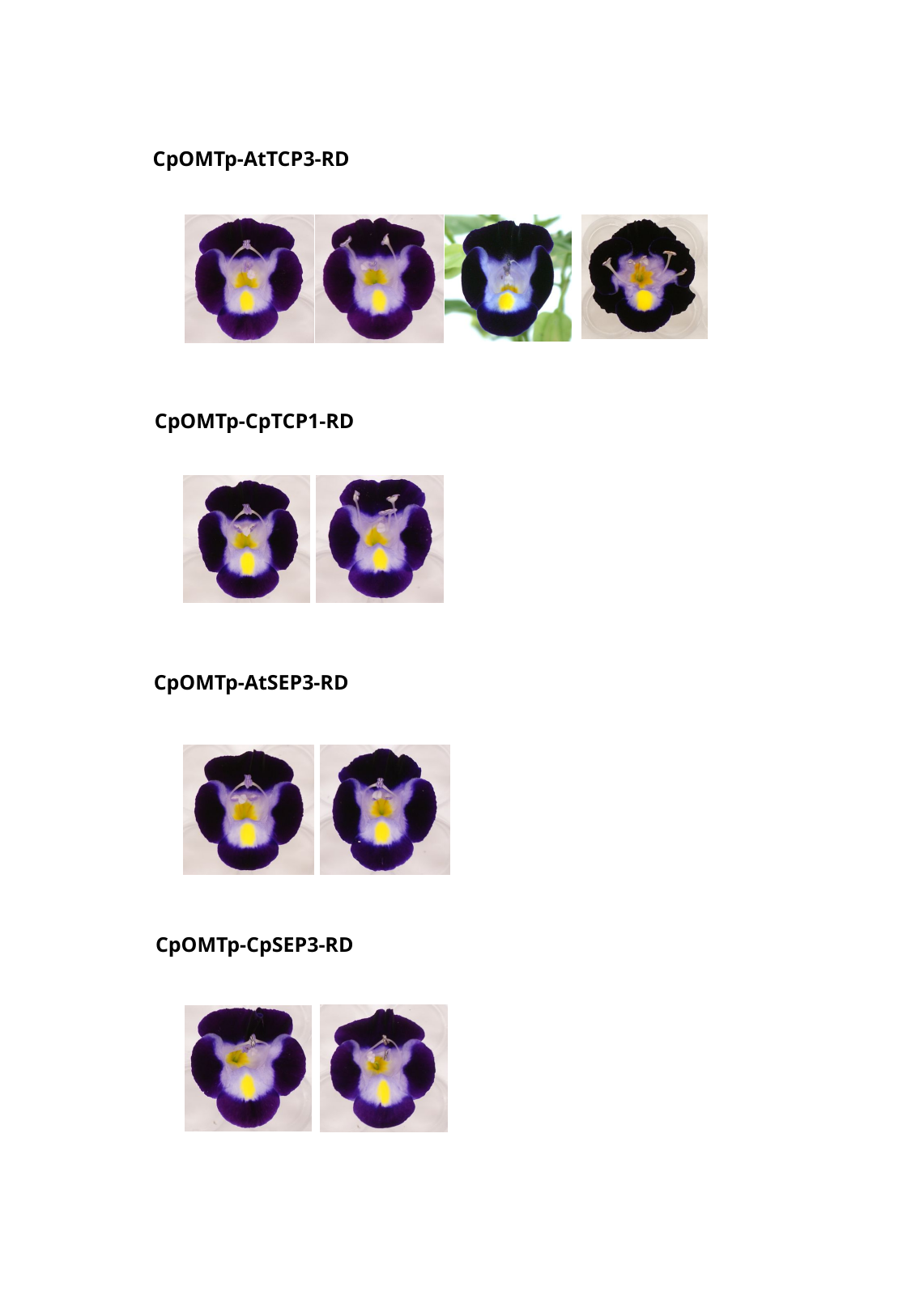

CpOMTp-AtTCP3-RD
CpOMTp-CpTCP1-RD
CpOMTp-AtSEP3-RD
CpOMTp-CpSEP3-RD
