# Supplementary material for: Combination of Cyclamen persicum Mill. floral gene promoters and chimeric repressors for the modification of ornamental traits in Torenia fournieri Lind
Source: Hortic Res. 2017 Mar 22;4:17008–. doi: 10.1038/hortres.2017.8 (PMC5386234; doi:10.1038/hortres.2017.8)
Supplement: Supplementary Figure 4 [file hortres20178-s4.ppt]

## Slide 1
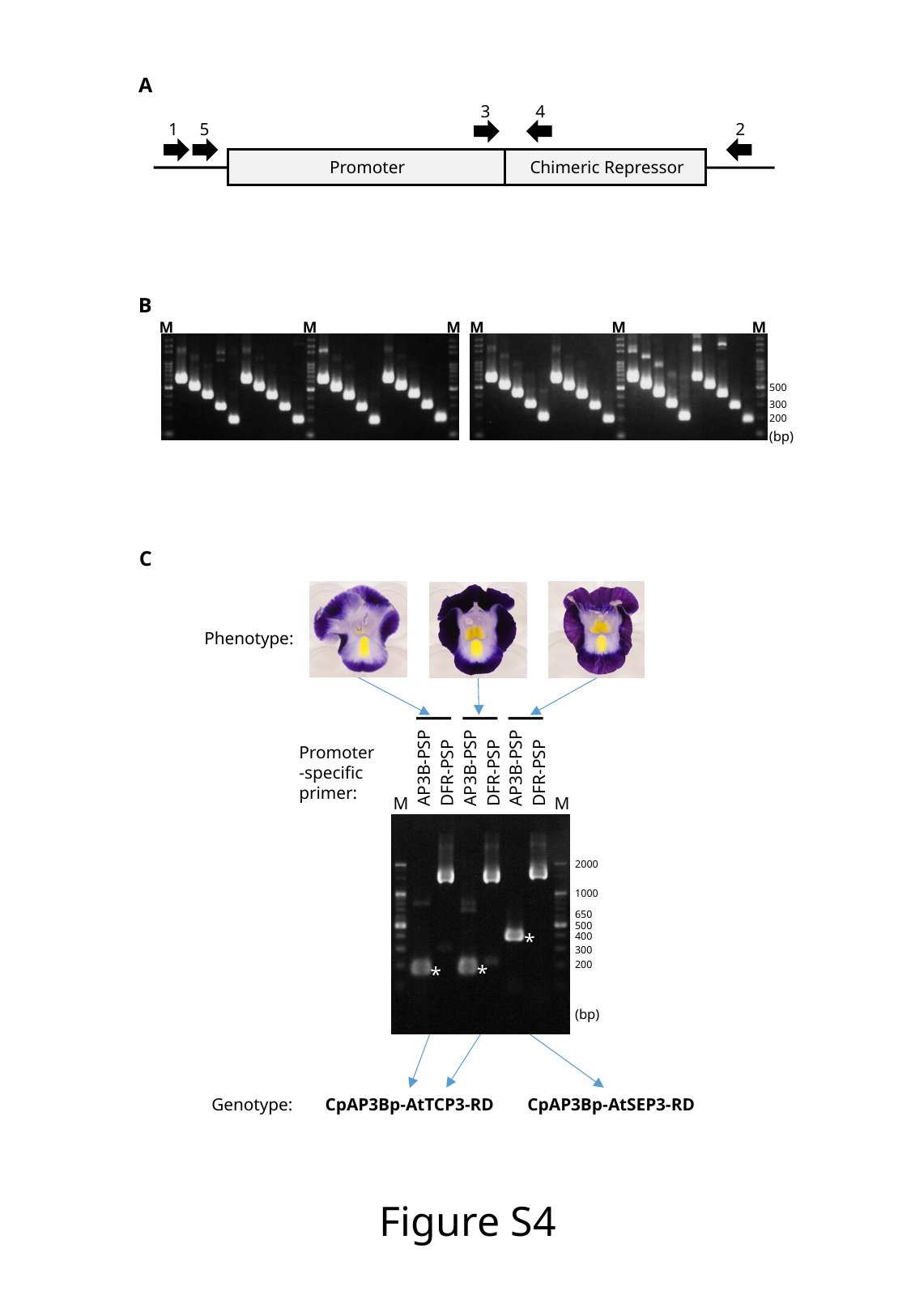

A
3
4
1
5
2
Promoter
Chimeric Repressor
B
M
M
M
M
M
M
500
300
200
(bp)
C
Phenotype:
AP3B-PSP
DFR-PSP
AP3B-PSP
DFR-PSP
AP3B-PSP
DFR-PSP
Promoter
-specific
primer:
M
M
2000
1000
650
500
*
400
300
200
*
*
(bp)
Genotype:
CpAP3Bp-AtTCP3-RD
CpAP3Bp-AtSEP3-RD
Figure S4
